# The knowledge, attitude, and practices of food handlers in central South African hospices

**DOI:** 10.1002/fsn3.1499

**Published:** 2020-05-12

**Authors:** Jane Sebolelo Nkhebenyane, Ryk Lues

**Affiliations:** ^1^ Department of Life Sciences Central University of Technology Bloemfontein South Africa

**Keywords:** food handlers, food safety training and food safety knowledge, hospices

## Abstract

The escalating number of foodborne diseases and food poisoning outbreaks demand a better call for improved food‐handling practices. Hospices are typically described as nongovernmental organizations that offer palliative care to terminally ill patients. The majority of hospice food handlers are not trained in food safety aspects, and services are offered on a voluntary basis. In this study, a descriptive survey design comprising of semistructured questionnaire was utilized to assess the knowledge, attitudes, and practices of the hospice food handlers (*n* = 100) in hospices around Central South Africa. More than half of the participants (68%) had not taken basic food safety training. The average percentage of the correct answers on the knowledge questionnaire was 66.8%. The participants had a mean age of 35 years (*SD* = 9.27). Attendance of food safety course had a significant effect on both the practices of using gloves to touch or distribute unwrapped foods (*χ* ^2^ = 8.411, *p*‐value = .012), and washing hands after using gloves (*χ* ^2^ = 12.560, *p*‐value = .001). The overall KAP mean score was 78.38. A statistically significant difference was found between the trained and untrained food handlers regarding food safety knowledge (*p* < .001). There was substantial lack of knowledge regarding the correct temperature for a refrigerator including hot ready‐to‐eat food.

## Graphical abstract

Hospices are typically described as nongovernmental organizations that offer palliative care to terminally ill patients. The majority of hospice food handlers are not trained in food safety aspects, and this descriptive survey found that there was substantial lack of knowledge regarding the correct temperature for a refrigerator including hot ready‐to‐eat food.

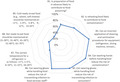

## 1 INTRODUCTION

Foodborne diseases continue to be a problem that contributes to morbidity and mortality across the globe. According to WHO (2015), the rate of foodborne diseases has been on the rise recently, and this has a negative influence on the health and economy aspect of developing countries more than those of developed countries. They mainly consist of a considerable variety of illnesses that become apparent after the ingestion of contaminated foods and food products. The etiological agents of foodborne diseases can be due to a variety of microbial pathogens, chemicals, and parasites that contaminate food at different points in the food production and preparation process. Both developed and developing countries are affected, and every individual in the world is exposed to the risk of contracting a foodborne disease (Fletcher, McLaws, & Ellis, 2013). Moreover, they constitute a common public health problem worldwide, but are generally under‐reported and poorly investigated in South Africa. Foodborne diseases may originate in the kitchen environment during food preparation and storage. For the purpose of this study, hospices are defined as nongovernmental organizations that offer palliative care to terminally ill patients (Gwyther et al., 2018). They usually do not receive funding from government and therefore operate under budgetary constraints for operational costs. Unfortunately, hospice kitchens are generally ill‐equipped in this healthcare setting as, unlike hospital kitchens, they are multipurpose areas for food preparation and storage and patients often have access to these kitchens. This creates a risk potential for food contamination as a range of microorganisms gain entry and spread to food, ultimately resulting in illness. Meals that are prepared and consumed by patients in hospices should thus receive special attention because the food is intended for a population group that is immune‐compromised. Many foods that are introduced into the domestic and hospice kitchens are frequently contaminated with naturally occurring pathogenic microorganisms. For example, *Staphylococcus aureus* is rated as the third most important cause of foodborne disease in the world (Normanno et al., 2005; Sani & Siow, 2014). It is well known that the primary reservoir of staphylococci in humans is the anterior nares, but the hands have also been found to harbor these bacteria in abundance. Moreover, hand contamination of food handlers with *S. aureus* has been identified as a critical risk factor for staphylococcal food poisoning (Ho, Boost, & O'Donoghue, 2015). In the current study, the respondents generally demonstrated a dearth of knowledge regarding the refrigeration temperature and the correct temperatures for hot and cold ready‐to‐eat foods. Against this background, the aim of the study was to evaluate the knowledge, attitudes, and practices of hospice food handlers in a selected area in South Africa. Demographic and work‐related factors associated with unsafe food‐handling practices were also evaluated.

## 2 MATERIALS AND METHODS

### 2.1 Research design

The study was conducted in fifteen hospices involving 100 food handler participants across central South Africa. A descriptive survey design was utilized to assess the knowledge, attitudes, and practices of the hospice food handlers (*n* = 100) that participated in the study. To determine construct and content validity, three food safety experts reviewed the questionnaire before administration and a pilot study was also undertaken to address any concerns associated with the questionnaire. A semistructured questionnaire comprising of multiple‐choice questions prepared and modified upon previously used questionnaires in other studies of similar nature (Buccheri et al., 2007) and the five keys to safer food of the World Health Organization (WHO, 2006) was administered to food handlers in the participating hospices. The questionnaire contained five sections: (a) demographic characteristics and employment status, (b) knowledge pertaining to food hygiene, (c) attitudes related to the prevention of foodborne diseases, (d) procedures to be utilized toward foodborne disease prevention, and (e) information sources regarding food hygiene. Knowledge questions related to foodborne disease agents and foods that are epidemiologically associated with pathogen transmission highlighted notable microorganisms from which the participants had the following options to select: yes, no, and don't know, concerning their possible association with foodborne disease, and further link one food vehicle to each pathogen. Responses were deemed to be right only when a food item commonly known to be a vehicle for the pathogen concerned was selected. Regarding the responses for the practice category, the following options were given: always, often, and occasionally. The food handlers in the hospices are often volunteers with a limited knowledge of food storage and preparation in the compromised health context. They are responsible for cooking and serving meals and cleaning the premises.

### 2.2 Sampling protocol and sampling sites

Hospices accommodating HIV patients were selected from the Hospice Palliative Care Association (HPCA) list. A purposive sampling methodology was utilized. There are approximately 120 registered hospices in South Africa of which about 20 have inpatient units and food preparation areas. Accommodation in hospices with an inpatient section is usually offered in converted houses or buildings allocated for this purpose by means of social grants and sponsorships. Such units generally accommodate 4–8 staff members comprising of a registered nurse or nurses, administrative and support staff, and food handlers. Patient number ranges from 10 to 30. Of the identified hospices, nine (100%) were located in the Free State province, three in the Eastern Cape province, and three in the Northern Cape province. All the food handlers working at the selected facilities at the time of the study were recruited, averaging just over six individuals per hospice. Permission was requested from and granted by all the managers of the targeted hospices to administer the semistructured questionnaire to their food‐handling staff, and a 100% participation rate of handlers at the selected facilities was achieved. Where the need arose to repeat the survey, this was done after consultation and with the approval of the relevant respondent and facility manager. Prior to the completion of the questionnaires, the study background and aim were explained to the food handlers and their rights and role in the study were clarified. Given the demographic and socioeconomic standing of the hospice food handlers, it was considered best practice that the questionnaire be administered to the group (4–8 participants) at each facility in a selected venue in the presence of the principal researcher and trained assistants so that any uncertainties could be addressed. The completed questionnaires were then collected for analysis upon completion by the food handlers. The University of Free State Ethical Committee and the Hospice Board approved the study and granted ethical clearance UFS‐HSD2016/1088.

### 2.3 Data analysis

Questionnaires were manually coded in order to allow consequent data processing. The questionnaire contained five sections as described in Section 2.1, and there were 37 questions in total. The scores obtained by the food handlers on the food safety knowledge, attitude, and practice questions were determined based on the multiple‐choice response to each statement. A score of +1 was allocated when the right option had been marked, −1 was assigned if the wrong answer had been selected, and 0 was assigned if the “don't know/uncertain” option had been selected. The combined percentage score for the food handlers was then computed by dividing the score sum by the highest possible score. Statistical evaluation of association between questionnaire answers and demographic and work‐related attributes such as the length of service in hospice occupation was conducted by the classification of responses for each section as dichotomous variables: Knowledge was classified and noted as correct versus incorrect/unknown; attitudes as agreement versus disagreement/uncertain, and practices as safe when answered “always” (“occasionally” for the question D6) versus unsafe when answered “often” or “never” (“always” for the question D6). Data were analyzed by the SPSS software 14.0 version (SPSS, Inc.). Data were analyzed and reported using descriptive statistics (frequencies, means, and standard deviation). Since the scale of measurement used in the data collection process is categorical, the most appropriate test statistic to evaluate any relationships between measurements is the chi‐square test. Statistical differences were set at *p* < .05.

## 3 RESULTS

### 3.1 Participants' profile

In total, 100 hospice food handlers participated in the study. All the respondents were of female gender with a mean age of 35 years (*SD* = 9.27). The majority had completed Grade 8 (Table 1), while only 30% of the respondents had a Grade 10 to Grade 12 qualification. The mean length of service in employment was 4 years (*SD* = 2.67). The majority (68%) of the food handlers had received no training in food safety, and only 32% of the respondents had attended one formal training course on food hygiene aspects.

**Table 1:** Demographic characteristics of interviewed food handlers (*n* = 100) in 15 hospices

Characteristics of survey respondents		Mean (*SD*)
Age (year)		
<35	56	35 ± 9.27
≥35	44	
Length of service in employment (year)		
<4	57	4 ± 2.67
≥4	43	
Education level (grade)		
<10	30	10 ± 2.17
≥10	70	
Attendance of at least one course on food hygiene		
Yes	32	
No	68	

#### 3.1.1 Food handler knowledge

**Figure 1:**
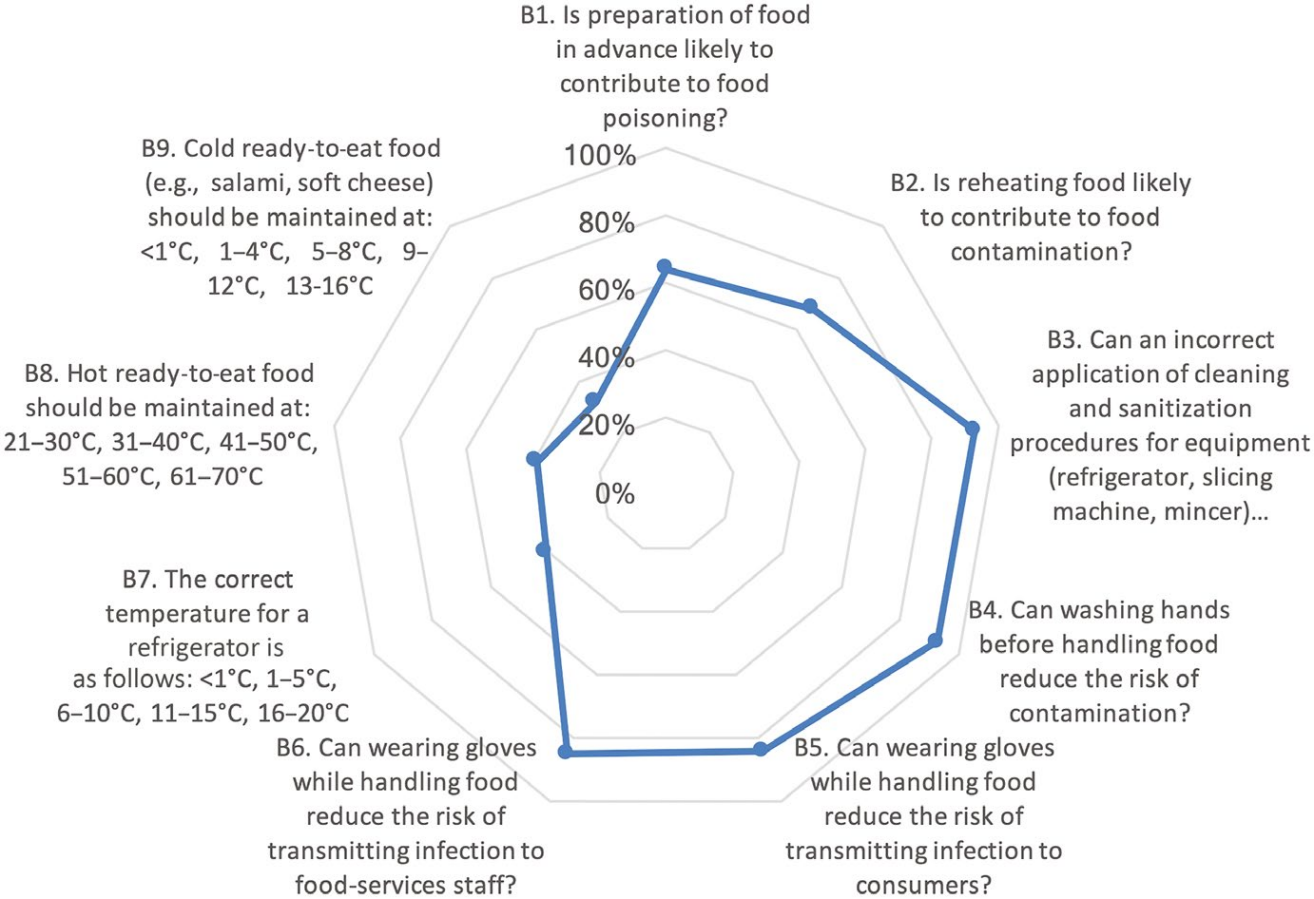
Knowledge of food preparation and/or preservation, hygiene standards, and transmission of foodborne diseases

The survey of the food handlers' knowledge demonstrated that 66.8% of the answers were correct. The most noteworthy findings for this section are outlined in Figure 1. The categories on which respondents had the highest level of knowledge are shown to be above the 80% contour line on the spiderweb graph. The overall KAP mean score was 78.38. The food handlers had very good knowledge of cleaning and sanitization procedures of equipment, washing hands before handling food, and wearing gloves while handling food. For all these mentioned variables, the food handlers obtained more than 80%. However, there was substantial lack of knowledge regarding the correct temperature for a refrigerator, hot ready‐to‐eat food including cold ready‐to‐eat food. The majority (64%) of the respondents knew that preparation of food in advance may pose the risk of food poisoning, while 68% was aware of the risks associated with reheating dishes before meal consumption. Moreover, 93% of the food handlers concurred that requisite cleaning and sanitization processes are partly responsible for the control and prevention of foodborne hazards. Almost all the respondents (93%) knew that washing hands before handling food decreases the risk of contamination. The majority (84%) of the food handlers also had the knowledge that using gloves while handling food has the potential to decrease the risk of infection transmission to consumers. However, only 41% of the food handlers had knowledge of the proper working temperature of a refrigerator, while 59% had no knowledge of the correct refrigeration temperature.

#### 3.1.2 Factors affecting the knowledge of food preparation and/or preservation, hygiene standards, and transmission of foodborne diseases

The study evaluated the effects of demographic variables on food handlers' knowledge of food preparation, preservation, hygiene standards, and the transmission of foodborne diseases. As the scale of measurement used in the data analysis process was categorical, the most appropriate test statistic to evaluate any relationships between measurements was the chi‐square test. With reference to items B1–B11, the responses “I don't know” and “No” were combined because very few chose the “I don't know” option. This was done to make the computation of the chi‐square test easier and more valid.

#### 3.1.3 Effects of age on the knowledge of food preparation and/or preservation, hygiene standards, and transmission of foodborne diseases

The data presented in Table 2 reveal that age had a notable effect on the knowledge of food poisoning (*χ* ^2^ = 17.686, *p*‐value = .005) and that food preparation in advance could be a contributing factor in foodborne pathogen transmission. Just more than 50% (10 out of 19) of the food handlers in the 25‐year age category indicated that preparation of food in advance was likely to contribute to food poisoning. The highest proportion of respondents who indicated that the practice of preparing food in advance had the potential to be partly responsible for food poisoning was in the 26–40 age category.

**Table 2:** Effects of age on the knowledge of food hygiene and transmission of diseases (only significant relationship shown)

Variables	A1. Age group							Fisher's exact chi‐square tests	
	Up to 25 years	26–30 years	31–35 years	36–40 years	41–45 years	46–50 years	Above 50 years	Statistic	*p*‐value
B1. Is preparation of food in advance likely to contribute to food poisoning?									
Yes	10	16	16	11	8	0	3	17.686	.005
No	9	4	2	7	6	5	3		
B2. Is reheating food likely to contribute to food contamination?									
Yes	13	14	16	12	10	2	1	12.145	.047
No	6	6	2	6	4	3	5		
B8. Hot ready‐to‐eat food should be maintained at: 21–30, 31–40, 41–50, 51–60, and 61–70°C									
Yes	5	9	8	6	10	1	0	12.317	.047
No	14	11	10	12	4	4	6		

#### 3.1.4 Effects of length of service on food preparation knowledge and/or preservation, hygiene standards, and transmission of foodborne diseases

The data presented in Table 3 show that length of service had a significant effect on the knowledge that incorrect cleaning and sanitization of equipment increase the risk of transmitting foodborne diseases to consumers (*χ* ^2^ = 14.629, *p*‐value = .000). The table also indicates that, in all the different service length categories, 100% of the respondents indicated that inappropriate utilization of cleaning and sanitization processes of equipment (refrigerator, slicing machine, mincing machine) has the potential to increase the transmission of foodborne diseases to consumers. The exception was the 1–2 years' service category as 39% in this category was unaware of this fact. The percentage obtained in this study for the cleaning and sanitation procedure is much higher than that obtained by Bolton, Meally, Blair, McDowell, and Cowan (2008) wherein only 35% of the respondent had knowledge on sanitation aspects. The data also showed that length of service had a significant effect on the knowledge that the practice of wearing gloves while handling food decreases the risk of transmitting foodborne infection to patients (*χ* ^2^ = 11.689, *p*‐value = .009). Length of service was also associated with knowledge of correct refrigeration temperature (*χ* ^2^ = 16.251, *p*‐value = .002). For example, 90% of the 3–4 years' service respondents knew the correct temperature for the refrigeration of food. Education level was also significantly associated with the knowledge that reheating food may contribute to food contamination (*χ* ^2^ = 10.427*, p*‐value = .049). Approximately 37% of the Grade 12 education‐level category knew that reheating food was likely to contribute to contamination. A study (Martins, Hogg, & Otero, 2012) also found that participants who have completed Grade 4 (primary school), and 6th year of formal education, had scores that were statistically significantly different from those who completed the 12th year. This may be attributed to the fact that food hygiene information is implicit in various subjects in the secondary school such as the compulsory Natural Sciences Learning Area in Grades 8–9 and Life Orientation, which is compulsory for all learners up to Grade 12 level.

**Table 3:** Effects of length of service and education on the knowledge of food hygiene and transmission of diseases

Variables	A2. Length of service					Fisher's exact chi‐square tests	
	<1 year	1–2 years	3–4 years	5–6 years	6+ years	Statistic	*p*‐value
B3. Can an incorrect application of cleaning and sanitization procedures for equipment (refrigerator, slicing machine, mincer) increase the risk of foodborne disease to consumers?							
Yes	12	18	38	14	11	14.629	.000
No	0	7	0	0	0		
B6. Can wearing gloves while handling food reduce the risk of transmitting infection to food‐services staff?							
Yes	10	16	36	12	11	11.689	.009
No	2	9	2	2	0		
B7. Select the correct refrigeration temperature by indicating “yes” or “no” on the following options: <1, 1–5, 6–10, 11–15, 16–20°C							
Yes	10	5	18	6	2	16.251	.002
No	2	20	20	8	9		

#### 3.1.5 Effects of attendance of food hygiene courses on the knowledge of food preparation and/or preservation, hygiene standards, and transmission of foodborne diseases

The results shown in Table 4 suggest that attendance of courses carries a significant effect on the knowledge that preparation of food in advance is likely to be partly responsible for food poisoning (*χ* ^2^ = 6.474, *p*‐value = .011), and the correct refrigerator temperature (*χ* ^2^ = 11.797, *p*‐value = .001).

**Table 4:** Effects of attendance of courses on food hygiene on the knowledge of food hygiene and transmission of diseases

	A4. Attended courses on food hygiene		Fisher's exact chi‐square tests	
	Yes	No	Statistic	*p*‐value
B1. Is preparation of food in advance likely to contribute to food poisoning?				
Yes	26	38	6.474	.011
No	6	30		
B7. Select the correct refrigeration temperature by indicating “yes” or “no” on the following options: <1, 1–5, 6–10, 11–15, and 16–20°C				
Yes	21	20	11.797	.001
No	11	48		
B8. Hot ready‐to‐eat food should be maintained at: 21–30, 31–40, 41–50, 51–60, and 61–70°C				
Yes	25	14	30.280	.000
No	7	54		
B9. Cold ready‐to‐eat food (e.g., salami, soft cheese) should be maintained at: <1, 1–4, 5–8, 9–12, and 13–16°C				
Yes	18	14	12.718	.000
No	14	54		

### 3.2 Food handler attitude

**Figure 2:**
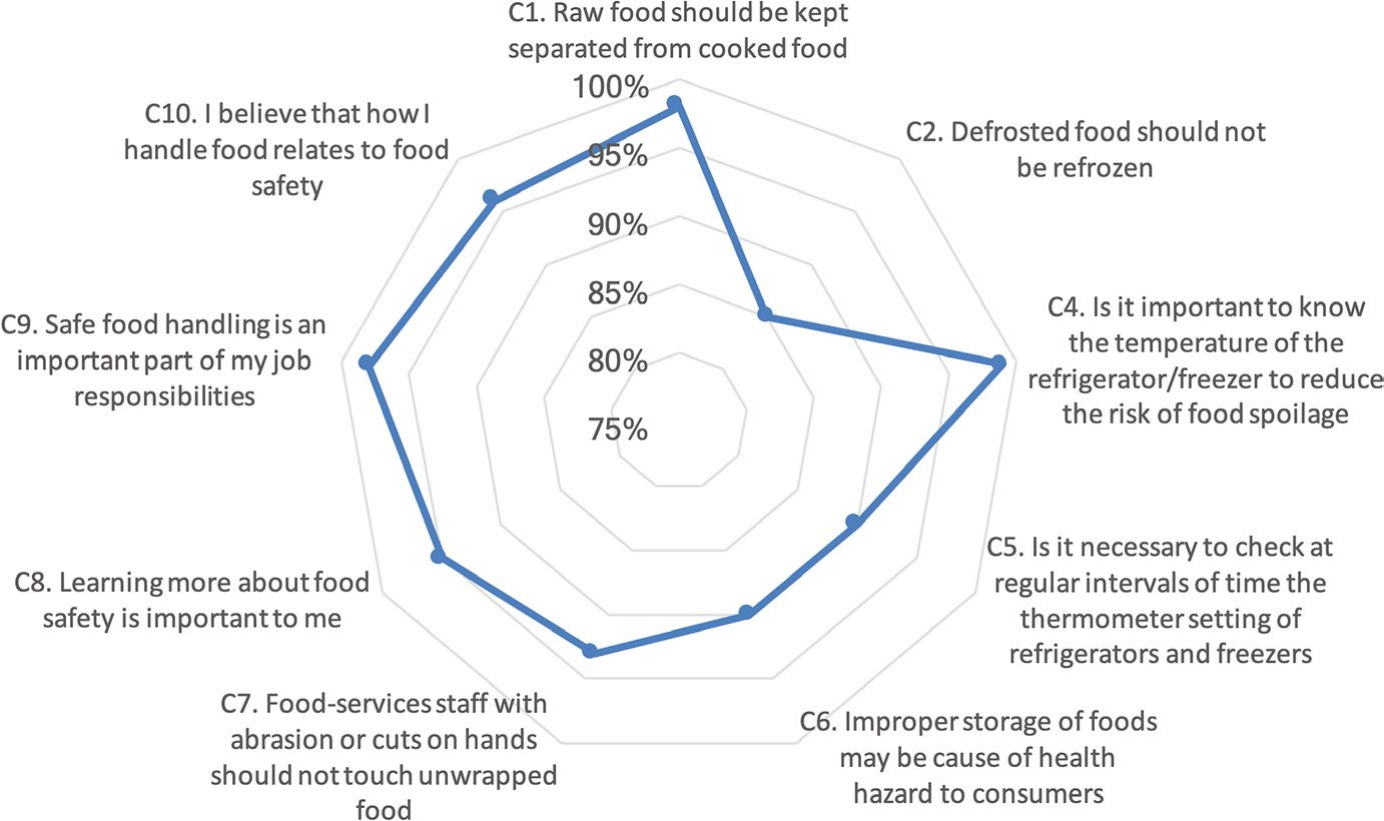
Attitudes toward food hygiene and related issues

The most noteworthy responses for this section are illustrated in Figure 2. Almost all the food handlers (92%) were in agreement that uncooked food should be kept separate from the food that is cooked, and only about 8% did not feel the obligation to embrace this key measure, to prevent cross‐contamination. Also, the majority of respondents (85%) were aware that defrosted food should not be refrozen. The use of caps, masks, and protective gloves including adequate clothing was well supported by the respondents as 94% of the respondents agreed and recognized the importance of wearing personal protective garments during food preparation. The percentage obtained in this study is much higher compared with that of Akabanda, Hlortsi, and Owusu‐Kwarteng (2017), wherein 60% of the respondents highlighted that wearing caps, masks, and protective gloves, and appropriate clothing can reduce the possible risk of food contamination. The positive response in the current study was encouraging because a reduction in incidences of foodborne diseases is strongly influenced by the attitudes of food handlers toward the implementation of food safety requirements. The study also found that the majority (90%) of the respondents agreed that it was essential to inspect food preparation and storage equipment at regular intervals. Almost all the respondents (90%) agreed that improper food storage temperatures might compromise the health of consumers. The majority of the respondents (93%) also concurred that abrasions or cuts on food handlers' hands pose a health risk and that food handlers should ideally not have any contact with unwrapped food. On the aspect of learning more about food safety, an overwhelming majority (98%) supported increasing their knowledge of food‐handling practices to ensure food safety. Age and length of service also had a significant impact on the attitude that defrosted food should not be refrozen (*p*‐values = .002 and .005, respectively).

### 3.3 Food handler self‐reported practices

The responses for this section are displayed in Table 5. Only 62% of the respondents reported that they always use gloves when they touch or distribute unwrapped foods, while 71% reported washing hands before using gloves. The majority (79%) of the respondents reported washing hands after using gloves, while 73% reported that they use protective clothing when touching or distributing unwrapped foods.

**Table 5:** Descriptive statistics of practices

Attitudes toward food hygiene and related issues	Occasionally	Often	Always
E1. Do you use gloves when you touch or distribute unwrapped foods?	21%	17%	62%
	21%	17%	62%
E2. Do you wash your hands before using gloves?	4%	25%	71%
	4%	25%	71%
E3. Do you wash your hands after using gloves?	10%	10%	80%
	10%	10%	80%
E4. Do you use protective clothing when you touch or distribute unwrapped foods?	20%	7%	73%
	20%	7%	73%
E5. Do you use a mask when you touch or distribute unwrapped foods?	29%	24%	47%
	29%	24%	47%
E6. Do you wear a cap when you touch or distribute unwrapped foods?	15%	15%	70%
	15%	15%	70%
E7. Do you wash your hands before touching unwrapped raw foods?	1%	15%	84%
	1%	15%	84%
E8. Do you wash your hands before touching unwrapped cooked foods?	1%	8%	91%
	1%	8%	91%
E9. Do you wash your hands after touching unwrapped cooked foods?	2%	5%	93%
	2%	5%	93%

#### 3.3.1 Effects of age on practices

Age had a significant effect (*p* = .038) on the practice of using protective clothing to touch or distribute unwrapped foods. About 15% of the age groups 26–30 and 36–40 indicated that they always use the supplied protective clothing when touching or distributing unwrapped foods, while about 5% of the age group 46–50 indicated “always” regarding this practice.

#### 3.3.2 Effects of attendance of courses on practices

The results that are presented in Table 6 show that attendance of courses has a significant effect on both the practices of using gloves to touch or distribute unwrapped (Chi‐square = 8.411, *p*‐value = .012), and washing hands after using gloves (*χ* ^2^ = 12.560, *p*‐value = .001). A low number (26%) of trained food handlers indicated that they “always” use gloves when touching or distributing unwrapped foods, while 36% of untrained food handlers indicated “always” for the same practice. Only about 2% of trained food handlers indicated that they “occasionally” use gloves when touching or distributing unwrapped foods, while the untrained group who applied this practice was 19%. Research has indicated that food safety training has the potential to prevent or curb food contamination risks as improved knowledge encourages food handlers to adjust and advance their skills. However, it has been emphasized (Acikel et al., 2008; Campos et al., 2009) that such training should be accompanied by regular inspection of the activities of the workers involved. None of the trained food handlers indicated “often” or “occasionally” in terms of the practice of washing hands after using gloves, whereas 10% of the untrained cohort indicated that they “occasionally” washed their hands after using gloves. However, 48% of the participants who never attended a course on food hygiene indicated that they “always” washed their hands after using gloves. The WHO (2009) recommends that hand hygiene must be performed immediately after glove removal to prevent further dissemination and transmission of microorganisms. Additionally, it has been stated by Todd, Michaels, Greig, Smith, and Bartleson (2010) that when gloves are used correctly, this practice can substantially reduce opportunities for food contamination. Another significant effect was the practice of using protective clothing when touching or distributing unwrapped foods (*χ* ^2^ = 14.698, *p*‐value = .001) and that of using a mask when touching or distributing unwrapped foods (*χ* ^2^ = 10.053, *p*‐value = .006). Lastly, there was also a significant difference (*χ* ^2^ = 5.953, *p*‐value = .026) on the practice of washing hands before touching unwrapped foods.

**Table 6:** Effects of attendance of courses on practices

Practices	A4. Attended courses on food hygiene		Fisher's exact chi‐square tests	
	Yes	No	Statistic	*p*‐value
E1. Do you use gloves when you touch or distribute unwrapped foods?				
Occasionally	2	19	8.411	.012
Often	4	13		
Always	26	36		
E3. Do you wash your hands after using gloves?				
Occasionally	0	10	12.560	.001
Often	0	10		
Always	32	48		
E4. Do you use protective clothing when you touch or distribute unwrapped foods?				
Occasionally	0	20	14.698	.001
Often	3	4		
Always	29	44		
E5. Do you use a mask when you touch or distribute unwrapped foods?				
Occasionally	2	27	13.532	.001
Often	9	15		
Always	21	26		
E6. Do you wear a cap when you touch or distribute unwrapped foods?				
Occasionally	0	15	10.053	.006
Often	5	10		
Always	27	43		
E8. Do you wash your hands before touching unwrapped cooked foods?				
Occasionally	1	0	5.953	.026
Often	0	8		
Always	31	60		

## 4 DISCUSSION

As alluded to earlier, majority of South African hospice food handlers do not receive formal food safety training. They rely mainly on demonstrations by supervisors in order to execute their duties. However, a small percentage of hospice managers expose their food handlers to food safety training. This study revealed a knowledge gap among the participating food handlers with regard to the optimum refrigeration temperature when storing food. This finding is similar to Dora‐Liyana, Mahyudin, Ismail‐Fitry, Ahmad‐Zaki, and Rasiyuddin (2018), where food handlers were found to be less familiar with time and temperature abuse. Hence, it is not surprising that hospice food handlers demonstrated an apparent lack of knowledge on temperature control (Figure 1). To elaborate further, a study conducted by Marais, Conradie, and Labadarios (2007) showed that the least successfully answered questions concerning food safety were related to temperature control. To support this, a study (Webb & Morancie, 2015), showed that knowledge of critical temperatures was inadequate among food handlers. In the current study, only about 39% and 32% seemed to be well versed in the proper holding temperatures of hot and cold ready‐to‐eat food, respectively. This particular knowledge gap in a hospice could possibly be attributed to insufficient information on this important food safety control measure while more emphasis is placed on personal hygiene. More than half of the food handlers were not mindful of the relationship between cholera and food as only 41% checked the correct answer in the questionnaire. The possible explanation for this could be that water is most commonly known as a vehicle for cholera. This finding is similar to that obtained by Liu et al. (2015), where food handlers demonstrated poor knowledge of foodborne pathogens. It is a well‐known fact that food preparation in advance could possibly promote food poisoning. However, Table 2 indicates that all the respondents in the age group 46–50 indicated that the practice that relates to food preparation well in advance is unlikely to be instrumental to food poisoning. This finding is contrary to the results obtained by Martins et al. (2012), wherein it was highlighted that the older age group had more advanced knowledge of this aspect of food preparation than their younger counterparts. In the current study, it was also determined that age had a significant effect on the knowledge of whether reheating food is likely to contribute to food contamination (*χ* ^2^ = 12.145, *p*‐value = .047) and the temperature at which hot ready‐to‐eat food should be sustained (*χ* ^2^ = 12.317, *p*‐value = .047). Surprisingly, it was also interesting to note that none of the older age group (>50 years) had knowledge of the correct holding temperature of hot ready‐to‐eat food. A study (Baş, Ersun, & Kivanç, 2006) found that almost 70% of food poisoning outbreaks were mainly caused by time and temperature abuse and cross‐contamination. Many studies have documented the benefits of regularly attending food safety training to achieve effective performance and modify knowledge. However, other authors are not in agreement that training and knowledge of the food handlers alone will inevitably improve practice (Da Cunha, Stedefeldt, & De Rosso, 2014). In this study, approximately 30% of the food handlers who have not attended any food safety course believed that preparation of food in advance is unlikely to contribute to food poisoning (Table 4). Preparation of food in advance is necessary in some of the hospices depending on the size of the establishment, and such food has to enter the cold chain immediately to be reheated later for menu items. Of more concern for immunocompromised people is the risk of microbial growth on ready‐to‐eat foods such as sprouts (*Escherichia coli*), deli meats and soft cheeses (listeria), shellfish (vibrio), and raw milk (*Salmonella* spp., *Campylobacter jejuni*, *S. aureus*). Moreover, studies (Bou‐Mitri, Mahmoud, El Gerges, & Jaoude, 2018; Liz Martins & Rocha, 2014) have shown that preparing food in advance, using incorrect thawing methods, and improper food‐holding temperatures are some of the contributing factors to foodborne outbreaks. The working environment of hospice food handlers can be stressful at times and requires the ability to multitask, but this practice is a potential hazard for microbial growth in food as food handlers' attention to storage conditions and wearing protective clothing and accessories may be distracted. Food handlers who are not trained and do not possess enough knowledge regarding the abuse of time and temperature and the effects thereof on food safety can potentially cause disease outbreaks among already health‐compromised patients. Other studies have also reported a similar finding; for example, Walker, Pritchard, and Forsythe (2003) and Akabanda et al. (2017) found that shortage of ample food handler knowledge regarding aspects of temperature control was common. In general, the current study determined that the attitude of food handlers toward food safety ranged from worrisome to satisfactory (Figure 2). For example, 5% of the age group (31–35 and 36–40) and 50% of the age group (>50) were of the same opinion that defrosted food should not be refrozen. Conversely, a study (Buccheri et al., 2007) revealed that a significant proportion of respondents (86.8%) were unaware that defrosted food should not be refrozen. Approximately 31% of the trained food handlers stated that they “always” washed their hands before touching unwrapped foods, while none of the same group stated “often” (Table 6). The results of this study are higher than those obtained by Bas, Ersun, and Kıvanc (2004), where only 5% of the food handlers reported that they “always” wash hands prior to touching the foods that are not wrapped. In the current study, only 8% of the untrained food handlers stated that they “often” washed their hands when touching unwrapped food. This finding is not encouraging due to the risk factors associated with ineffective hand washing. The limitation of the study is that data are self‐reported.

## 5 CONCLUSIONS

Food hygiene and food safety are of paramount importance in a hospice setting, especially as microbial hazard reduction is vital in a context where health‐compromised patients are treated. Undeniable evidence exists that the number of foodborne diseases has increased recently in the world, particularly in developing countries (Trepka, Murunga, Cherry, Huffman, & Dixon, 2006). However, the latter authors argue that nearly all of these cases of foodborne diseases could have been prevented if food protection approaches had been followed from production to consumption. The kitchen is generally the point of origin of food contamination that occurs during food preparation, handling, and storage processes. Food can be mishandled at several points in this chain, and therefore, knowledge of the correct handling of food at all stages of the preparation and storage processes is essential in eradicating incidences of foodborne disease outbreaks. The current study revealed that food handlers in the hospice context had positive attitudes toward and generally effective practices of food safety. However, the rate of only 50% who supported the use of masks when touching or distributing unwrapped food should be addressed. It was also evident that food safety knowledge regarding temperature control was inadequate, and this underscores the need for food safety training with emphasis on refrigeration and freezing temperatures. This proposal is supported by the fact that 61% and 68% of the food handlers had no knowledge of the correct holding temperatures of hot and cold ready‐to‐eat food, respectively. This finding raises concerns because temperature is a vital variable in food safety practices, especially concerning meals that are prepared for immune‐compromised patients. Other studies such as those by Angelillo, Foresta, Scozzafava, and Pavia (2001) and Askarian, Gholamhosein, Aminbaig, Memish, and Jafari (2004) also highlight that knowledge shortfalls regarding critical temperatures in hospital settings should be addressed in the training of food handlers. Because food producers cannot guarantee the absence of pathogens in the food they supply, food handlers' role regarding the prevention of foodborne diseases should never be underestimated. Hospice food handlers should be mindful of the important role of personal hygiene as a critical component toward the prevention of food contamination and dissemination of gastroenteric illnesses. This is especially true concerning the fact that some enteric pathogens such as *E. coli* 0157:H7 have a low minimum infective dose. The necessary association of positive behavior, attitudes, and continued education of food handlers to understand the need for the sustainability of safe food‐handling practices has been emphasized and should never be underestimated (Gomes‐Neves, Araújo, Ramos, & Cardoso, 2007; Howes, McEwen, Griffiths, & Harris, 1996). The meals prepared and distributed in hospices should be accorded special attention because they are intended for a vulnerable group with a high risk for contracting several diseases due to microbial pathogens that may be present in the food. Hospices should establish and standardize regulations for food handlers in order to ensure high‐quality meals with minimal bacterial contamination potential. The results of the study thus underscore a pressing need for food safety education for all hospice food handlers.

## CONFLICT OF INTEREST

The authors declare that they have no conflicts of interests.

## ETHICAL APPROVAL

The University of Free State Ethical Committee and the Hospice Board approved the study and granted ethical clearance UFS‐HSD2016/1088. This study does not involve any human or animal testing.

## INFORMED CONSENT

Written informed consent was obtained from all study participants.
